# Pulmonary arteriovenous malformations: haemodynamics and shunt closure

**DOI:** 10.1007/s12471-014-0546-8

**Published:** 2014-03-26

**Authors:** D. Merkus

**Affiliations:** Department of Cardiology, Erasmus MC, PO Box 2040, 3000 CA Rotterdam, the Netherlands

The first description of pulmonary arteriovenous malformations (PAVM) was made already in 1897 by Churton [1]. PAVM are abnormal vascular structures that connect the pulmonary arterial bed directly to the pulmonary venous bed, thereby bypassing the pulmonary capillaries and resulting in an intrapulmonary shunt which, depending on number and size of the PAVM, may result in arterial desaturation and paradoxical systemic embolisation [2]. Because the PAVM are thin-walled vascular structures, they are prone to rupture, resulting in haemoptysis. A major step forward in the treatment of PAVM was the use of percutaneous transcatheter embolisation, using femoral venous access [3]. This procedure is safe and reduces the risk of paradoxical thromboembolisation and haemoptysis. Moreover, by reducing shunt flow, arterial oxygen saturation increases.

Although percutaneous closure of PAVM has been performed for years, and a surprisingly small effect on pulmonary haemodynamics has been shown [4], the study by Vorselaars and co-workers in the present issue of the Netherlands Heart Journal [5] is the first to investigate the immediate consequences of PAVM closure on systemic haemodynamics. Using Finapres technology, which derives changes in stroke volume and systemic pressure from the pressure wave form as measured on a finger, they describe an overall decrease in stroke volume and cardiac output that corresponds in magnitude with the overall decrease in shunt fraction. This decrease in stroke volume is similar to the decrease in stroke volume, as measured directly by right heart catheterisation, observed in a case study at 4 months follow-up after PAVM [6]. It was speculated that such a decrease in stroke volume helped to explain the absence of an increase in pulmonary artery pressures [4], despite the fact that pulmonary vascular resistance should obviously increase as a consequence of the closure of the low resistance PAVM.

The mechanism behind the observed decrease in stroke volume is unclear. It could be speculated that shunt closure and the accompanying increase in pulmonary vascular resistance result in a slight increase in right ventricular afterload, which limits right ventricular output. It is, however, more likely to assume that oxygen is regulated to fulfil the oxygen demand of peripheral tissues; shunt closure increases the oxygen content of arterial blood and hence for the same oxygen delivery, in the presence of improved oxygenation, less flow is required. The latter explanation is in accordance with the observation that the shunt fraction prior to embolisation (14 %) is similar in magnitude to the decrease in cardiac output (10 %) [5].

As pulmonary artery pressure increases during exercise, the driving pressure for the shunt flow increases, resulting in an increased shunt flow, which is accompanied by augmented arterial desaturation during exercise [7–9]. However, exercise capacity is surprisingly well-maintained in patients with PAVM, potentially due to the capability of the right heart to deal with volume overload. Indeed, although most patients report an increased exercise capacity and quality of life following shunt closure, an objective increase in exercise capacity is not found in all patients [10]. It is possible that changes in pulmonary and systemic haemodynamics upon shunt closure, which are relatively small under resting conditions, are exacerbated during exercise. Thus, while the study by Vorselaars in the present issue of the Netherlands Heart Journal provides an important observation in resting patients [5], a comprehensive evaluation of shunt flow, pulmonary and systemic haemodynamics and oxygen saturation during exercise prior to and following shunt closure would be of great benefit to enhance our understanding of the implications of shunt closure on exercise capacity.

## References

[1] Churton T (1897). Multiple aneurysm of pulmonary artery. Br Med J (Clin Res Ed).

[2] Cartin-Ceba R, Swanson KL, Krowka MJ (2013). Pulmonary arteriovenous malformations. Chest.

[3] Chilvers ER, Whyte MK, Jackson JE (1990). Effect of percutaneous transcatheter embolization on pulmonary function, right-to-left shunt, and arterial oxygenation in patients with pulmonary arteriovenous malformations. Am Rev Respir Dis.

[4] Shovlin CL, Tighe HC, Davies RJ (2008). Embolisation of pulmonary arteriovenous malformations: no consistent effect on pulmonary artery pressure. Eur Respir J.

[5] Vorselaars VMM, Velthuis S, Mager JJ (2014). Direct hemodynamic effects of pulmonary arteriovenous malformation embolism. Neth Heart J.

[6] Andrivet P, Lofaso F, Carette MF (1995). Haemodynamics and gas exchange before and after coil embolization of pulmonary arteriovenous malformations. Eur Respir J.

[7] Li W, Niu B, Henderson K (2011). Reproducibility of oxygen saturation monitoring during six-minute walk test and exercise stress test in patients with pulmonary arteriovenous malformations associated with hereditary hemorrhagic telangiectasia. Pediatr Cardiol.

[8] Whyte MK, Hughes JM, Jackson JE (1993). Cardiopulmonary response to exercise in patients with intrapulmonary vascular shunts. J Appl Physiol.

[9] Whyte MK, Peters AM, Hughes JM (1992). Quantification of right to left shunt at rest and during exercise in patients with pulmonary arteriovenous malformations. Thorax.

[10] Gupta P, Mordin C, Curtis J (2002). Pulmonary arteriovenous malformations: effect of embolization on right-to-left shunt, hypoxemia, and exercise tolerance in 66 patients. AJR Am J Roentgenol.

